# Protocol for a Systematic Review of Interventions Not Delivered by Speech and Language Therapists for Children With Speech Sound Disorders

**DOI:** 10.1111/1460-6984.70187

**Published:** 2026-01-21

**Authors:** Maria Viliam Cairney, Sarah Kevill, Joanne Cleland

**Affiliations:** ^1^ University of Strathclyde Glasgow UK

**Keywords:** communication skills, indirect interventions, speech sound disorders, speech therapy, systematic review

## Abstract

**Background:**

Children with speech sound disorders (SSDs) of unknown origin usually need high‐intensity speech intervention delivered by a speech and language therapist (SLT) and there is a rich evidence base focusing on these interventions. However, access to direct SLT services can be challenging, leaving many children with no timely support. To date there is no systematic review of possible interventions for children with SSD of unknown origin that do not require direct SLT input.

**Aims:**

To describe a protocol for a systematic review of non‐SLT‐delivered interventions for children with SSD of unknown origin.

**Methods and procedures:**

A systematic literature review will be performed following the preferred reporting items for systematic reviews and meta‐analyses (PRISMA) guidelines, by two academic SLTs who are topic experts and a subject librarian. The search will include electronic databases (including ASSIA, CENTRAL, CINAHL, The Cochrane Library, Embase, LILACS, MEDLINE, APA PsychInfo, PubMed, SCI, Scopus, SSCI) for peer reviewed studies published in English with no limits on publication date. Study selection will follow pre‐specified inclusion criteria: majority children with SSD of unknown origin; and exclusion criteria: interventions targeting signing, AAC, language or delivered by SLTs. The remaining studies will be assessed for risk of bias. Data will be extracted using comprehensive structured forms. A narrative and quantitative synthesis will be provided.

**Conclusions and implications:**

The results of this review will support SLTs to make evidence‐based decisions when supporting children with SSDs of unknown origin while managing long waiting lists and large caseloads. The systematic review will make recommendations for improved service delivery, suggesting whether clinical pathways for this client group may also include interventions not involving SLTs.

**Trial registration:**

This systematic review protocol has been registered with PROSPERO, registration number CRD420251006629.

**WHAT THIS PAPER ADDS:**

*What is already known on this subject*

- There is a wealth of information about the evidence base for interventions for children with speech sound disorders (SSDs) of unknown origin delivered directly by speech and language therapists (SLTs). However, there is limited information about the effectiveness of alternative interventions delivered by therapy partners or mediated via a digital tool.

*What this study adds to the existing knowledge*

- This paper outlines the protocol for a systematic literature review on interventions for children with SSD that are not delivered by SLTs.

*What are the potential or actual clinical implications of this study?*

- The results of this review will provide SLTs with the current evidence base for interventions for children with SSD that do not require their direct input, which they might consider for implementation while managing large caseloads or long waiting lists.

## 1 Introduction

### 1.1 Rationale

Speech sound disorder (SSD) is an umbrella term describing challenges with producing or perceiving speech sounds (Stringer et al. 2023). It affects approximately 3.4%–3.8% of school aged children, suggesting that around one child in a classroom of 30 will require clinical support (Eadie et al. 2015; Shriberg et al. 1999; Wren et al. 2016). SSDs can arise from differences in anatomy or physiology, for example due to conditions such as cleft palate ± lip (CP ± L), or they can be of (currently) unknown cause, in which case the cause may be functional (Cleland et al. 2025). SSDs of unknown cause comprise the largest proportion of cases (Shriberg et al. 2019). In both cases, SSDs can occur alongside language, literacy and other learning differences (Hayiou‐Thomas et al. 2017).

Timely intervention from speech and language therapists (SLTs) is needed for children with SSDs. Research suggests that children with persistent SSD after the age of 8 are likely to underperform compared to peers (controlling for verbal IQ and performance IQ) in core academic subjects as late as 14 years of age (Wren et al. 2021). To be effective, SLT interventions for children with SSDs need to be provided at a high intensity (Hegarty et al. 2018; Leafe et al. 2024). A high intensity is considered a dose of at least 50 practice trials for 30 therapy sessions (Williams 2012), although in their review of the evidence Leafe et al. (2025) suggest it should be considered 70–100 trials. This is higher than what is reported as current practice in the United Kingdom where most SLTs reported being able to elicit up to 30 trials per session, across 9–12 sessions (Hegarty et al. 2018) and what is practiced worldwide (Leafe et al. 2025). Unfortunately, this is not possible in all clinical settings and families report that they are struggling to access services in some countries (McGill et al. 2021, McGill et al. 2020).

There are reports on worldwide demands for SLT services, and unmet needs for people with speech, language and communication needs (SLCN), linked to staff shortages, long waiting lists, or due to a lack of people practising the SLT profession in multiple countries (Chittem et al. 2022; Kamenov et al. 2019; Law and Conti‐Ramsden 2000; McGill et al. 2021, McGill et al. 2020; Oh 2019; Tan 2015; Wylie et al. 2023). The lack of SLT services in some areas, can be illustrated for example by Tinney et al. (2007) who identified only one speech therapist in Ghana, Bunning et al. (2014) who reported no SLTs in rural Kenya, and Khan et al. (2015) who identified no SLT services in Madagascar. Even in countries where the profession is well established there can be challenges meeting population needs. In the United Kingdom, data from community services in National Health Service (NHS) England in February 2024 showed that there were 72 661 children on waiting lists for SLT intervention, of whom 23 387 were waiting for up to a year (NHS England 2025). This means that there may be a key period in a child's life (e.g., primary school education) where they are not able to access SLT services, which are needed to facilitate effective communication of their needs and thoughts, making friends and accessing the curriculum, potentially leading to stigma and isolation (McCormack et al. 2010; McLeod et al. 2013; Nilsson et al. 2021; Wren et al. 2021).

In this context, in addition to taking action to increase investment in SLT services, it is important to consider what alternative interventions are available to put preventative and supportive measures in place for underserved families, either while on waiting lists, or in conjunction with direct therapy from an SLT. A public health approach aims to achieve disease prevention on a population level, using three levels of prevention with increasing levels of impact (Public Health Scotland 2024).

However, in practice, public health programmes are rarely funded to an extent that can serve the entire population (universal interventions), despite evidence that increased spending in this area is ultimately cost‐effective (World Health Organization 2012). Hence, government programmes often target subpopulations at higher risk (targeted interventions) (Dodge 2020). These approaches targeting whole populations or subpopulations are contrasted with a specialist level of intervention, which requires an individual to be named on a clinical caseload, and would be considered ‘specialist care’ (Dodge 2020; Ebbels et al. 2019). In the SLT literature Ebbels et al. (2019) discuss the terms universal, targeted and specialist interventions and existing ambiguities. They propose that if the care is individualised to a specific child, for whom an SLT has duty of care, it should be considered specialist level of intervention, regardless of whether it is delivered directly by an SLT or by another person. This definition will be followed in this paper.

Public health is an important area within the remit of SLTs (Enderby and Law 2019). The balanced tiered approach of combining universal and targeted interventions has been adopted as part of the evidence based pathways available for children with Developmental Language Disorder (DLD)—another condition that requires high intensity of specialist intervention for children of a similar age group as those with SSD of unknown origin (Ebbels et al. 2019; Frizelle et al. 2021). DLD can have a lifelong impact on an individual's communication and DLD of unknown origin has a higher prevalence (7.58%) than SSDs of unknown origin (Norbury et al. 2016). The International Classification of Functioning, Disability and Health supports an approach that targets activity and participation, not just via medical‐model intervention of body structures and functions (e.g., specialist level speech intervention) but also through environmental adaptations that may include shifting societal attitudes (potentially a targeted or universal intervention) (McLeod and Bleile 2004). A holistic approach to care is consistent with the preferences expressed by families of children with CP ± L in Williams et al. (2021). Those families and children cared about improving independence, making friends, developing skill at sports and schoolwork and not just improvements in speech intelligibility or articulation.

Typically, SSDs of unknown origin are considered in the context of tertiary prevention or specialist care with limited examples of interventions designed to be delivered by non‐SLTs or not targeting speech (Cleland, and Stringer 2025). This can be explained because, as discussed earlier, the current evidence base supports high intensity specialist interventions (despite these not being feasible in many understaffed contexts). In addition, while there has been relatively little research on how the public accesses SLT services (Law et al. 2013), and despite developments to expand the public health roles of SLTs particularly in the context of language development, there is still limited evidence of the effectiveness of these approaches, with reports limited to mainly the United Kingdom, United States and Australia (Smith et al. 2017) and anecdotally few SLTs have public health as the sole focus of their role. Here it is important to specify that there is a difference in how the terms direct and indirect therapy are used in the literature. For example, Pickstone et al. (2009) use them to refer to interventions where the child is or is not present. Others use them to refer to interventions that may not be delivered directly by a SLT but where an SLT may have a consultative role (Boyle et al. 2009; Williams et al. 2021). To avoid inconsistencies, these terms will be avoided in the present paper.

Wren et al. (2018) carried out a systematic review and classification of interventions targeting the speech of pre‐school children with SSD of unknown origin. Twenty‐five studies met their quality inclusion criteria, of which only one focused on an environmental intervention (Yoder et al. 2005). That study reported that parental recasting was associated with improved intelligibility. In addition, Harding et al. (2024) conducted an umbrella review of outcome measures, assessments and interventions for children with SSD of unknown origin, identifying 46 interventions. Of them there was one type of intervention that specifically emphasised the inclusion of non‐SLTs but was still led by one: “Parents and Children Together” (PACT) (Bowen and Cupples 1999). While Harding et al. (2024) did not specifically exclude non‐SLT‐led interventions they may not have been available for inclusion due to specificities of their methodology (an umbrella review) and search strategy (they did not include key words specifically targeting these types of interventions).

Therefore, the present review aims to complement the existing reviews by investigating interventions for children with SSD that are not primarily delivered by an SLT and that focus on children with SSD of unknown origin. In addition, the interventions do not have to focus on speech, but may instead target other areas that are within the remit of SLTs, such as social communication and participation, advocacy, quality of life, emotional regulation and well‐being, which do not necessarily aim to alleviate the speech impairment but support the child holistically. The aim of this review is to provide an overview of interventions that can support children with SSD holistically (not just focusing on their impairment) that do not require the involvement of SLTs.

#### 1.1.1 Reviews to Date

There are no systematic reviews investigating existing interventions not delivered by SLTs, including, parent‐led, teacher‐led, peer‐ and self‐led interventions, as well as computer‐based interventions and environmental or public health interventions for children with SSDs.

### 1.2 Objective

This study aims to investigate what interventions are available for children with SSD of unknown origin that are not led by a SLT.

## 2 Methods

This systematic review and classification of interventions will follow the recommendations of the PRISMA 2020 statement (Page et al. 2021).

### 2.1 Eligibility Criteria

#### 2.1.1 Study Characteristics

To capture a wide range of studies, including those at a lower stage of evidence, studies with all designs, from all contexts and in all settings and time frames will be included, although if too many relevant results emerge (>1000 results for initial screening), this may be narrowed down to the last 10 years, to manage workload and ensure a timely output. The remaining study characteristics are listed, following the PICO approach.

##### 2.1.1.1 Population

Studies will be included if they focus on children who are diagnosed with SSD, which is of unknown origin (inclusive of articulation disorder, phonological disorder/delay, childhood apraxia of speech, formerly known as developmental verbal dyspraxia) and at least 80% of the sample is of children from 2;00 until 17;11 at the start of intervention or at recruitment. Children with SSD will be included if they have co‐occurring language difficulties. Dysarthria will be excluded because it is usually associated with a known origin, such as Cerebral Palsy. Studies will be excluded if they focus on: 1. Children whose speech is developing typically; 2. Children with voice or fluency disorders; 3. Children with SSD associated with other conditions, such as learning disabilities, hearing impairment, Autism, CP±L, congenital or acquired neurological conditions (e.g., Cerebral Palsy), or genetic syndromes.

##### 2.1.1.2 Intervention

Studies will be included if they include the following targets or formats, which may be delivered individually or in groups:

1. Environmental interventions targeting the communication approaches of the child's family, friends, school and other communication partners;
2. Advocacy support;
3. Parent (carer)‐mediated interventions;
4. Peer‐mediated interventions;
5. Teacher‐mediated interventions;
6. Assistant‐led interventions;
7. Self‐led interventions;
8. Digitally led interventions (including smartphone applications, computer games, automated SMS‐consultations, online resources);

Studies will be included in the review even if they do not specify if the ultimate aim is for these interventions to be delivered in a targeted or universal model of delivery (i.e., to groups where individual children are not named on an SLT's caseload).

Studies will be excluded if they focus on:

1. Interventions requiring sign language training, Makaton training, Singalong training;
2. Augmentative and alternative communication interventions,
3. Language support,
4. Group therapy for children with SSD, led by SLTs;
5. SLTs working directly with a child in person or online

##### 2.1.1.3 Comparator

No comparison criteria are applied because we are including studies with varied designs.

##### 2.1.1.4 Outcomes

Broad inclusion criteria are applied with respect to outcomes. Studies will be included if at least one of the outcome measures addresses the child's speech sounds, the quality of life of the child or adjacent people involved (e.g., family, teachers, peers), the child's activity and participation, communication, or interaction. Studies will also be included if they report results on a qualitative or quantitative change of the child's environment (e.g., physical environment, attitudes, support and relationships). Studies will be excluded if the outcome measures only focus on outcomes unrelated to communication, activity, participation, or quality of life, such as intervention acceptability or if they describe the development of an intervention without assessing its effects.

#### 2.1.2 Report Characteristics

The review will include only English‐language studies due to resource restrictions. To maintain a minimum standard of quality studies will be included if they are published in peer‐reviewed journals, without restriction as to the time of publication.

### 2.2 Information Sources

The main bibliographic databases searched will be ASSIA—Applied Social Sciences Index & Abstracts; CENTRAL—Cochrane Central Register of Controlled Trials; CINAHL—Cumulative Index to Nursing and Allied Health Literature; The Cochrane Library; Embase—Embase via Ovid; LILACS—Latin American and Caribbean Health Sciences Literature; MEDLINE via Ovid; APA PsycInfo; PubMed; SCI—Science Citation Index; Scopus; SSCI—Social Science Citation Index.

Other important or specialist bibliographic databases will be Sociological Abstracts; Social Services Abstracts; Educational Resource Information Center (ERIC) via EBSCO; Linguistics and Language Behavior Abstracts; British Education Index.

These sources were also used by Wren et al. (2018) and cover a broad range of journals pertaining to medicine, psychology, linguistics and the allied health professions. Where a potentially relevant review article cannot be retrieved, direct contact with the study authors will be made. The reference lists of all studies included in the final review will be screened to search for additional relevant studies.

### 2.3 Search Strategy

A search strategy was developed via an initial search of the CINAHL database based on consultation of the authors—two subject experts and a subject librarian. In addition, the search terms of two relevant reviews were consulted (Harding et al. 2023; Wren et al. 2018). The search terms were used to search the title and abstract describing the articles. Table 1 presents the full search strategy for CINAHL, including the limits applied. The search terms will be adapted for each database for the full review.

**TABLE 1:** Full search strategy for CINAHL.

1.	XB (“speech sound disorder*” OR “phonological disorder*” OR “articulation disorder*” OR “articulation impairment*” OR “phonological impairment” OR “speech delay*” OR “speech impairment*” OR “speech disorder*” OR “speech sound difficult*” OR “speech retard* OR ”speech problem*“ OR ”speech handicap*“ OR ”apraxia of speech“ OR ”developmental verbal dyspraxia“ OR ”verbal dyspraxia" OR dyspraxia)
2.	XB (child* or youth* or boy* or girl* or kid* or juvenil* or teenage* or adolescen* or “young person*” or “young people*” or toddler* or youth OR pediatric OR paediatric OR “school‐age*”)
3.	XB (“parent‐led” OR “parent‐mediated” OR “mother‐led”, “mother‐mediated”, “father‐led”, “father‐mediated” OR “family‐led” OR “family‐mediated” OR “sibling‐led” OR “sibling‐mediated” OR “self‐led” OR “self‐guided” OR “home‐based” OR “teacher‐led” OR “teacher‐mediated” OR “assistant‐led” OR “assistant‐mediated“ OR ”school‐based“ OR “school‐led” OR ”classroom‐based“ OR ”computer‐assisted“ OR “computer‐mediated” OR software OR “computer application” OR website OR online OR ”technology‐delivered" OR “SMS” OR “text messag*” OR “mobile phon*” OR “mobile app*” OR “phone* app*” OR game OR indirect)
4.	1. AND 2. AND 3.
Limits	Language (English)

### 2.4 Study Records

#### 2.4.1 Data Management

Search results will be exported from the databases and imported into EndNote to identify and filter out duplicates, as well as to carry out title, and abstract screening and full text eligibility evaluation.

#### 2.4.2 Selection Process

Two of the authors will complete a double‐blind screen of 20% identified papers on title level and an agreement rate calculated. Disagreements will be discussed until consensus is reached. Then the first author will evaluate all remaining abstracts against the inclusion and exclusion criteria. 100% of the resulting abstracts will be independently evaluated by two of the authors, following the same process where disagreements are found. After the abstract screening, all the remaining articles will have their full texts collated by the first author. Two of the authors will independently evaluate all remaining full texts for inclusion using EndNote. The authors will have a consensus meeting and record all decisions for exclusion against each article, which will be reported in the final literature review. The results of the search process will be reported in a PRISMA flow diagram (Page et al. 2021).

### 2.5 Data Extraction Process, Data Items

Data from the remaining papers will be extracted by the first author. The third author will independently extract data from 20% of the included studies, and the agreement rate will be reported.

Disagreements will be resolved via consensus meetings. An extraction form presented in Table 2 will be used to record the extracted data.

**TABLE 2:** Data extraction form. ICF codes provided where available.

Reference	
Source of funding	
Setting	Country of origin Language of intervention Intervention setting
Type and number of participants (therapy partners) per category	Child with SSD, Immediate family (parents/carers or siblings) (e310), Extended family (e315), Friends (e320) Acquaintances, peers, classmates, neighbours and community members (children and adults) (e325) People in positions of authority (teachers, schools staff, teaching or speech and language therapy assisstants) (e330) People in subordinate positions (may be removed during review process) (e335) Personal care providers and personal assistants (e.g., support workers, childcare providers) (e340) Strangers (e345) Health professionals (e355) Other professionals (e360) Other (further subcategories may emerge during the review process)
Demographic data about children with SSD	Age Biological sex Diversity characteristics Comorbidity SSD subtype Socio economic status
Intervention target	Articulation functions (b320) Mental function of sequencing complex movements (e.g. for childhood apraxia of speech) (b176) Regulation of emotion (b1521) Auditory perception (b1560) Communicating—receiving (d310‐d315) Communicating—producing (d330‐d331) Conversation (d350) Human‐made changes to environment (e.g., putting in place communication‐friendly environment in school) (e2) Support and Relationships (e.g., interpersonal communication strategies, not involving speech) (e3) Attitudes (e.g., advocacy) (e4) Services, Systems and Policies (education and training systems, services and policies) (e5)

Study design	
Intervention characteristics	Intervention type Intervention method Intervention delivered by Service delivery framework (e.g., tiered model, integrated pathways, multidisciplinary) Service delivery format
Therapeutic conten	Dose Frequency Method Duration
Assessments	Measurement instruments Schedule

Outcome metrics	
Analysis	
Key conclusion	
Biological sex	
Effect size(s) (Cohen *d* unless otherwise specified)	
PEDro‐P score or SCED score	

The extraction form was developed by the authors, based on related reviews (Harding et al. 2023; Wren et al. 2018). Due to the novel focus on environmental interventions, the type of participants and intervention targets were expanded to include relevant ICF categories, adapted for children, as per the most recent version of the ICF browser at the time of writing.^1^ “Domesticated animals” is a subcategory of “Support and Relationships” in the ICF. We considered retaining it for completeness, as animal‐mediated therapies have been known to be used to support social communication (Chitic et al. 2012). However, even if available, it is unlikely they would fall under the remit of an SLT, so we excluded it. The data extraction tool will be revised if necessary, during the extraction process, and any changes will be reported in the final review. If quantitative analyses were carried out and there is insufficient data to calculate an effect size, we will email the corresponding author to request access to their dataset.

### 2.6 Outcomes and Prioritisation

Following the ICF‐based framework for goal setting in therapy for children with SSD from McLeod and Bleile (2004), the outcomes sought here are holistic: body structures and functions (including speech sound production and perception, emotional well‐being), activity and participation (including communicative participation and intelligibility) and reporting observed changes within their environment. As environmental interventions aim to indirectly affect the well‐being of an individual both environmental changes and individual well‐being outcomes will be considered. Table 3 provides an exhaustive list of all outcomes that will be considered. Both quantitative and qualitative outcomes will be reported where available. Despite this diversity, as specified in our inclusion criteria, the studies need to report at least one measure of speech sounds.

**TABLE 3:** Study outcomes, considered in this review.

Primary outcomes: changes in the child(ren) with SSD	Activity and participation of child(ren) with SSD (including communication)
	Speech sound production and/or perception
	Quality of life of child(ren) with SSD (including mental health outcomes, if reported)
Secondary outcomes: changes in the environment	Human‐made changes to physical environment (e.g., putting in place communication‐friendly environment in school)
	Support and relationships (e.g., interpersonal communication strategies, not involving speech)—reported uptake of strategies, accurate use of strategies
	Attitudes—observed changes in attitudes
	Services, systems and policies (education and training systems, services and policies)—completed implementation of new systems, services and policies

### 2.7 Risk of Bias in Individual Studies

Risk of bias in individual studies will be assessed using the Physiotherapy Evidence Database (PEDro) scale (Perdices et al. 2009) for rating methodological quality for group study designs and using the SCED scale for single case studies (Tate et al. 2008). Both tools have been previously used to assess evidence in Speech and Language Therapy contexts (Korkalainen et al. 2023; Sugden et al. 2019).

### 2.8 Data Synthesis

Following the approach by Wren et al. (2018), where appropriate, effect sizes and standard errors will be calculated using:

‐ the Campbell Collaboration effect size calculator (https://www.campbellcollaboration.org/calculator/)
‐ single‐subject experimental designs will be assessed using improvement rate difference (Parker et al. 2011)

In cases of missing data, the authors will be contacted, and failing to obtain it, the resulting synthesis tables will reflect missing data. Data synthesis tables will also report any conversions required for synthesis.

In addition, a narrative synthesis of the key findings of the studies will be presented. A narrative analysis will be used to discuss potential sources of heterogeneity in the study results.

### 2.9 Meta Bias(es) and Confidence in Cumulative Evidence

The quality of the body of evidence will be assessed using the Grading of Recommendations Assessment, Development and Evaluation (GRADE) guidelines (Balshem et al. 2011), which requires evaluation of the following criteria: study design, risk of bias, inconsistency, indirectness, imprecision, publication bias, effect size, residual confounding analysed.

## 3 Brief discussion

SSDs of unknown origin contribute to a large proportion of the caseloads of SLTs and require a lot of intensive specialist level intervention. In the context of increasing demands for Speech and Language Therapy services which are often not met, it is important to evaluate what alternative support can be provided to children and families. Recent systematic literature reviews on SSD interventions (Harding et al. 2024; Wren et al. 2018) have identified a large variety of SLT‐mediated interventions focusing on speech, but due to the nature of their scope there remains a gap of knowledge about interventions not delivered by SLTs and interventions that support children holistically beyond their speech sound production.

This systematic review aims to address the gap by collating and discussing the available evidence of alternative SSD interventions, that might be family‐, teacher‐, assistant‐, peer‐ or self‐ mediated and delivered via another person or a physical or digital interface, such as a booklet, mobile application, or a website. We expect to provide a comprehensive qualitative synthesis of the available interventions, summarising their context and intensity of delivery, as well as targets and therapy partners. In addition, if available, we will provide a quantitative synthesis of the interventions’ effectiveness by summarising reported effect sizes, and improvement rates. A limitation of this review is the limitation to using studies only published in English.

## 4 Conclusion

We believe that this review will be valuable to clinicians managing large caseloads or long waiting lists. Increased awareness of potentially effective interventions that do not require the input of SLTs may support service managers to implement a more tiered and balanced approach to service delivery, similar to current practice for DLD (Ebbels et al. 2019). This review will complement the existing reviews which systematise SLT‐delivered interventions targeting speech sounds, which remain currently the best practice for supporting children with SSDs (Cleland and Stringer, in press).

## Conflicts of Interest

The authors have no competing interests to report.

## Endnotes

1 ICF 2017 – English. URL https://apps.who.int/classifications/icfbrowser/(accessed 3.14.25).
